# Anthrax Lethal Toxin Suppresses Murine Cardiomyocyte Contractile Function and Intracellular Ca^2+^ Handling via a NADPH Oxidase-Dependent Mechanism

**DOI:** 10.1371/journal.pone.0013335

**Published:** 2010-10-13

**Authors:** Machender R. Kandadi, Yinan Hua, Heng Ma, Qun Li, Shu-ru Kuo, Arthur E. Frankel, Jun Ren

**Affiliations:** 1 Center for Cardiovascular Research and Alternative Medicine, University of Wyoming College of Health Sciences, Laramie, Wyoming, United States of America; 2 Cancer Research Institute of Scott and White Memorial Hospital, Temple, Texas, United States of America; University of California, Berkeley, United States of America

## Abstract

**Objectives:**

Anthrax infection is associated with devastating cardiovascular sequelae, suggesting unfavorable cardiovascular effects of toxins originated from *Bacillus anthracis* namely lethal and edema toxins. This study was designed to examine the direct effect of lethal toxins on cardiomyocyte contractile and intracellular Ca^2+^ properties.

**Methods:**

Murine cardiomyocyte contractile function and intracellular Ca^2+^ handling were evaluated including peak shortening (PS), maximal velocity of shortening/ relengthening (± dL/dt), time-to-PS (TPS), time-to-90% relengthening (TR_90_), intracellular Ca^2+^ rise measured as fura-2 fluorescent intensity (ΔFFI), and intracellular Ca^2+^ decay rate. Stress signaling and Ca^2+^ regulatory proteins were assessed using Western blot analysis.

**Results:**

In vitro exposure to a lethal toxin (0.05 – 50 nM) elicited a concentration-dependent depression on cardiomyocyte contractile and intracellular Ca^2+^ properties (PS, ± dL/dt, ΔFFI), along with prolonged duration of contraction and intracellular Ca^2+^ decay, the effects of which were nullified by the NADPH oxidase inhibitor apocynin. The lethal toxin significantly enhanced superoxide production and cell death, which were reversed by apocynin. *In vivo* lethal toxin exposure exerted similar time-dependent cardiomyocyte mechanical and intracellular Ca^2+^ responses. Stress signaling cascades including MEK1/2, p38, ERK and JNK were unaffected by *in vitro* lethal toxins whereas they were significantly altered by *in vivo* lethal toxins. Ca^2+^ regulatory proteins SERCA2a and phospholamban were also differentially regulated by *in vitro* and *in vivo* lethal toxins. Autophagy was drastically triggered although ER stress was minimally affected following lethal toxin exposure.

**Conclusions:**

Our findings indicate that lethal toxins directly compromised murine cardiomyocyte contractile function and intracellular Ca^2+^ through a NADPH oxidase-dependent mechanism.

## Introduction

*Bacillus anthracis* (*B. anthracis*), a spore-forming, Gram-positive bacterium, has been widely known as the causative agent of anthrax [1]. Accidental or intentional exposure of *B. anthracis* spores by oral, cutaneous or pulmonary routes often triggers infection resulting in a high mortality rate associated with severe hypotension [2], [3], [4]. Treatment remedies such as administration of antibiotics, vaccination or antibody to the toxin have shown some promises in the clinical management of *B. anthracis* infection [1], [5]. However, patients with anthrax exposure develop refractory hypotension unresponsive to antibiotics, fluid, pressor and respiratory support [2], [6]. The lack of reproducible benefit from critical care intervention in these patients has spurred experimental studies to uncover the pathophysiology and molecular basis of anthrax shock and the associated organ complications.

*B. anthracis* vegetative bacteria secrete 3 proteins namely protective antigen (PA), lethal factor (LF) and edema factor (EF), forming anthrax lethal toxin (PA and LF) and anthrax edema toxin (PA and EF) [7]. EF and LF bind to the pore entrance to be translocated into cytosolic space. LF is a Zinc-metalloprotease which specifically cleaves the NH_2_-terminal of mitogen-activated protein kinase kinases (MEKs) resulting in inactivation of the kinases. EF is a calmodulin-dependent adenylyl cyclase to promote intracellular cAMP accumulation and the associated cellular responses [8], [9]. Up-to-date, the main toxic mechanism of anthrax is considered to be derived from the lethality of purified preparations of lethal and edema toxins in rodents [10], [11]. The toxicity of bacterial strain may be reduced by a thousand-fold with mutated or inactivated toxin genes [12] while animals may be protected from fatal *B. anthracis* infection by prophylactic treatment with antibodies or vaccines to anthrax toxins [13], [14]. One of the most devastating consequences for anthrax infection is anthrax shock associated with edema and hemorrhage, suggesting poor cardiovascular sequelae of lethal and edema toxins [1], [15]. Lethal toxin has been shown to trigger a significant reduction in ejection fraction, decreased myocardial contractility and diastolic dysfunction [1], [16], [17]. On the other hand, edema toxin leads to a significant reduction in left ventricular volume (preload) and cardiac output without overt change in ejection fraction or myocardial contractility [16], [17]. These findings favor a more prominent role of lethal toxin over edema toxin in compromised myocardial contractile function although the precise mechanism(s) behind lethal toxin-elicited cardiac dysfunction is largely unknown. Therefore, the aim of the present study was to elucidate the role of lethal toxin on cardiomyocyte contractile function and intracellular Ca^2+^ properties (both *in vitro* and *in vivo*) as well as the underlying mechanism of action in isolated murine cardiomyocytes. Given the notion that proteins affording protection against ROS may play an important role in cell survival to lethal toxin infection as evidenced by proteomic studies [18], we examined superoxide anion production and involvement of the main superoxide anion generating enzyme NADPH oxidase in the heart following lethal toxin exposure. Expression of the stress signaling molecules MEK1/2, p38 MAP kinase, ERK and JNK as well as the essential Ca^2+^ regulatory proteins were also evaluated in an effort to better understand the mechanism of action behind lethal toxin-induced cardiomyocyte mechanical and intracellular Ca^2+^ handling responses, if any.

## Materials and Methods

### Generation of lethal toxin

Recombinant PA, LF and EF were produced and purified as previously described [16], [17]. The toxins were stored at −80°C in phosphate buffered saline, pH 7.4 (PBS). Immediately prior to injection, toxin components were thawed and mixed in PBS.

### Lethal toxin treatment (in vivo and in vitro)

All animal procedures described here were in accordance with humane animal care standards outlined in the NIH Guide for the Care and Use of Experimental and were approved the University of Wyoming Animal Care and Use Committee (#A-3216-01). In brief, adult male C57BL/6J mice (5–6 months of age) were kept in our institutional animal facility under well-controlled conditions of temperature (22±2°C), humidity (55±5%) and a 12 h/12 h light-dark cycle with access to food and water *ad libitum*. For acute lethal toxin challenge *in vivo*, mice were injected intraperitoneally with anthrax lethal toxin (2 µg/g body weight, LF+PA at 1∶2 ratio) and were sacrificed under anesthesia (ketamine/xylazine: 3∶1, 1.32 mg/kg, i.p.,) at 2, 4 and 18 hrs after lethal toxin injection. The dosages of lethal toxin injection were chosen based on earlier report of overt myocardial dysfunction [15]. To evaluate the role of NADPH oxidase in anthrax lethal toxin-induced cardiac dysfunction, if any, a cohort of mice was fed the NADPH oxidase inhibitor apocynin (80 µg/ml) in drinking water for 6 days [19] prior to anthrax challenge (2 µg/g b.w., i.p. for 18 hrs). For *in vitro* lethal toxin exposure, freshly isolated murine cardiomyocytes were incubated with lethal toxin (0.05 – 50 nM) at 37°C for 2 hrs with or without preincubation of the NADPH oxidase inhibitor apocynin (100 nM).

### Isolation of murine cardiomyocytes

Cardiomyocytes were isolated as described previously. In brief, mice were anesthetized using ketamine and xylazine (3∶5, 1.32 mg/kg). Hearts were rapidly removed and perfused with oxygenated (5% CO_2_/95% O_2_) Krebs-Henseleit bicarbonate (KHB) buffer containing (in mM) 118 NaCl, 4.7 KCl, 1.25 CaCl_2_, 1.2 MgSO_4_, 1.2 KH_2_PO_4_, 25 NaHCO_3_, 10 HEPES, and 11.1 glucose. Hearts were then perfused with a Ca^2+^-free KHB containing Liberase Blendzyme 4 (Hoffmann-La Roche Inc., Indianapolis, IN, USA) for 20 min. After perfusion, left ventricles were removed and minced to disperse cardiomyocytes in Ca^2+^-free KHB buffer. Extracellular Ca^2+^ was added incrementally back to 1.25 mmol/l. Only rod-shaped myocytes with clear edges were selected for mechanical and intracellular Ca^2+^ transient studies. Cells were used within 6 hrs of isolation [20].

### Cell shortening/relengthening

Mechanical properties of cardiomyocytes were assessed using a SoftEdge MyoCam system (IonOptix Corporation, Milton, MA, USA). In brief, cardiomyocytes were placed in a chamber mounted on the stage of an inverted microscope (Olympus, IX-70) and superfused at 25°C with a buffer containing (in mM): 131 NaCl, 4 KCl, 1 CaCl_2_, 1 MgCl_2_, 10 Glucose and 10 HEPES, at pH 7.4. The cells were field stimulated with suprathreshold voltage at a frequency of 0.5 Hz, 3 msec duration, using a pair of platinum wires placed on opposite sides of the chamber and connected to an electrical stimulator (FHC Inc, Brunswick, NE,USA). The myocyte being studied was displayed on a computer monitor using an IonOptix MyoCam camera. An IonOptix SoftEdge software was used to capture changes in cell length during shortening and relengthening. Cell shortening and relengthening were assessed using the following indices: peak shortening (PS), maximal velocities of cell shortening and relengthening (± dL/dt), time-to-PS (TPS), and time-to-90% relengthening (TR_90_) [20].

### Intracellular Ca^2+^ fluorescence measurement

Myocytes were loaded with fura-2/AM (0.5 µM) for 10 min and fluorescence measurements were recorded with a dual-excitation fluorescence photo multiplier system (Ionoptix). Cardiomyocytes were placed on an Olympus IX-70 inverted microscope and imaged through a Fluor 40x oil objective. Cells were exposed to light emitted by a 75 W lamp and passed through either a 360 or a 380 nm filter, while being stimulated to contract at 0.5 Hz. Fluorescence emissions were detected between 480 and 520 nm by a photomultiplier tube after first illuminating the cells at 360 nm for 0.5 s then at 380 nm for the duration of the recording protocol (333 Hz sampling rate). The 360 nm excitation scan was repeated at the end of the protocol and qualitative changes in intracellular Ca^2+^ concentration were inferred from the ratio of fura-2 fluorescence intensity at two wavelengths (360/380). Fluorescence decay time was assessed as an indication of intracellular Ca^2+^ clearing. Single and bi-exponential curve fit was applied to calculate the intracellular Ca^2+^ decay constant [20].

### Intracellular fluorescence measurement of superoxide (O_2_^−^)

Intracellular O_2_^−^ was monitored by changes in fluorescence intensity resulted from intracellular probe oxidation [20]. In brief, lethal toxin-treated cardiomyocytes (with or without the NADPH oxidase inhibitor apocynin) were loaded with 5 µM dihydroethidium (DHE) (Molecular Probes, Eugene, OR, USA) for 30 min at 37°C and washed with PBS buffer. Cells were sampled randomly using an Olympus BX-51 microscope equipped with an Olympus MagnaFire™ SP digital camera and ImagePro image analysis software (Media Cybernetics, Silver Spring, MD, USA). Fluorescence was calibrated with InSpeck microspheres (Molecular Probes). An average of 100 cells was evaluated using the grid crossing method in 15 visual fields per isolation.

### MTT assay for cell viability

[3-(4,5-Dimethylthiazol-2-yl)-2,5-Diphenyltetrazolium Bromide] (MTT) assay is based on transformation of the tetrazolium salt MTT by active mitochondria to an insoluble formazan salt. Lethal toxin-treated cardiomyocytes (with or without the NADPH oxidase inhibitor apocynin) were plated in microtiter plate at a density of 3×10^5^ cells/ml. MTT was added to each well with a final concentration of 0.5 mg/ml, and the plates were incubated for 2 hrs at 37°C. The formazan crystals in each well were dissolved in dimethyl sulfoxide (150 µl/well). Formazan was quantified spectroscopically at 560 nm using a SpectraMax® 190 spectrophotometer [21].

### Cell culture and treatment

H9c2 cells, a clonal cell line derived from fetal rat hearts, were purchased from American Type Culture Collection (ATCC, Manassas, VA, USA). Cells were grown in Dulbecco's modified Eagle's medium (DMEM) supplemented with 10% fetal bovine serum (FBS) (Gibco, Grand Island, NY, USA) and 1% penicillin and streptomycin and maintained in 95% air and 5% CO2 at 37°C. Cells were grown to 80% confluence before incubation with anthrax lethal toxin.

### LC3B-GFP-adenovirus production and infection

Adenovirus containing LC3-GFP construct (kindly provided by Dr. Cindy Miranti from Van Andel Institute, Grand Rapids, MI, USA) was propagated using HEK293 cell line. In brief, cells were infected with LC3-GFP adenovirus. Upon plaque formation, infected cells were collected, washed with PBS, resuspended in culture medium and lysed by three cycles of freezing (dry ice)-thawing (37°C). Cell debris was collected by centrifugation and aliquots of supernatant with viral particles were stored at −80°C. Adenovirus was purified using an Adeno-X Maxi purification kit (Clontech Laboratories, Inc. Mountain View, CA, USA). H9c2 cells were grown to confluence on Lab-Tek chamber slide. Cells were then infected at an MOI of 2 with adenoviruses expressing LC3-GFP fusion protein. Medium was replaced with fresh DMEM after 6 hrs. Twenty four hrs later, cells were visualized for autophagy using fluorescence microscopy.

### Quantification of the GFP-LC3

H9c2 cells transfected with GFP-LC3 adenovirus were treated with or without anthrax lethal toxin. The cells were fixed with 4% paraformaldehyde in PBS for 20 min at room temperature. Cells were washed with PBS three times. Cover slips were mounted on the slides using Vecta mountTM AQ-aqueous mounting medium (Vector Laboratories, Inc, Burlingame, CA, USA). For autophagy assessment, cells were visualized at 40× magnification using a Olympus BX51fluorescence microscope (Olympus America, Inc, Melville, NY, USA) and the percentage of cells showing numerous GFP-LC3 puncta (>10 dots/cells) were scored as described previously [22]. A minimum of 75–100 cells were scored for each condition in at least three independent experiments.

### Western blot analysis

Expression of the stress signaling molecules MEK1/2, ERK, JNK and p38 MAP kinase, the intracellular Ca^2+^ regulatory proteins sarco(endo)plasmic reticulum Ca^2+^-ATPase (SERCA2a) and phospholamban, the ER stress proteins BIP (GRP78), pan and phospho-eIF2α, the autophagy markers Beclin-1 and LC3B were assessed. In brief, left ventricular tissue was sonicated in a lysis buffer containing (in mmol/l): Tris 10, NaCl 150, EDTA 5, 1% Triton X-100 and protease inhibitor cocktail followed by centrifugation at 12,000×g for 10 min. Equal amount (30 µg) protein and prestained molecular weight marker were separated using the 7%–12% SDS-polyacrylamide gels in a minigel apparatus (Mini-PROTEAN II, Bio-Rad, Hercules, CA, USA), and were then transferred to nitrocellulose membranes (0.2 µm pore size, Bio-Rad). Membranes were blocked for 1 hr in 5% nonfat milk before being rinsed in TBS-T. The membranes were incubated overnight at 4°C with anti-MEK1/2 (1∶1,000, Cell Signaling Technology, Beverly, MA, USA), anti-phospho-MEK1/2 (Ser217/221, 1∶1,000, Cell Signaling), anti-ERK (1∶1,000, Santa Cruz Biotechnology, Santa Cruz, CA, USA), anti-phospho-ERK (Thr202, 1∶1,000, Santa Cruz), anti-JNK (1∶1,000, Cell Signaling), anti-phospho-JNK (Thr183/Ttyr185, 1∶1,000, Cell Signaling), anti-p38 MAP kinase (1∶1,000, Cell Signaling), anti-phospho-p38 MAP kinase (Thr180/Tyr182, 1∶1,000, Cell Signaling), anti-eIF2α (1∶1,000, Cell Signaling), anti-phospho-eIF2α (1∶1,000, Cell Signaling), anti-BIP (GRP78, 1∶1,000, Cell Signaling), anti-Beclin-1 (1∶1,000, Cell Signaling), anti-LC3B (1∶1,000, Cell Signaling), anti-Bcl-xL (1∶1,000, Cell Signaling), anti-Bcl-2 (1∶1,000, Santa Cruz), anti-Bax (1∶1,000, Cell Signaling), anti-Bad (1∶1,000, Cell Signaling), anti-cytochrome-C (1∶1,000, Cell Signaling) and anti-caspase-12 (1∶1,000, Cell Signaling) antibodies. After incubation with the primary antibodies, blots were incubated with horseradish peroxidase-linked secondary antibodies (1∶5,000) for 60 min at room temperature. Immunoreactive bands were detected using the Super Signal West Dura Extended Duration Substrate (Pierce, Milwaukee, WI, USA). The intensity of bands was measured with a scanning densitometer (Model GS-800; Bio-Rad) coupled with a Bio-Rad personal computer analysis software. α-Tubulin was used as the loading control [20].

### Data Analysis

Data were presented as MEAN ± SEM. Statistical significance (p<0.05) for each variable was estimated by analysis of variance (ANOVA).

## Results

### In vitro effect of lethal toxin on cardiomyocyte shortening and intracellular Ca^2+^ transients

**Figure 1:**
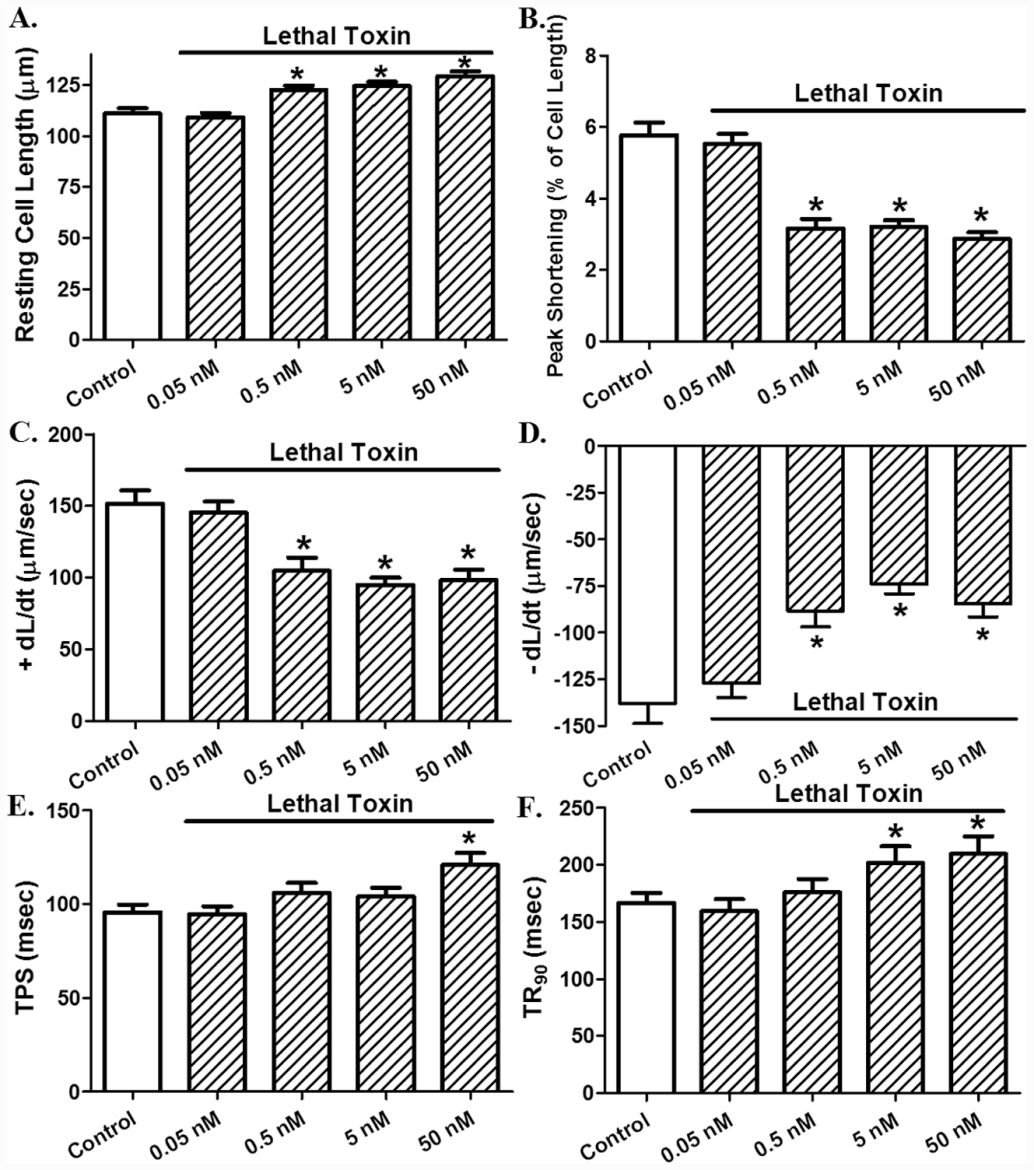
Effect of *in vitro* lethal toxin (0.05-50 nM, 2 hrs) exposure on cardiomyocyte contractile function. A: resting cell length; B: Peak shortening (normalized to cell length); C: Maximal velocity of shortening (+ dL/dt); D: Maximal velocity of relengthening (- dL/dt); E: Time-to peak shortening (TPS); and F: Time-to-90% relengthening (TR_90_). Mean ± SEM, n = 95-101 cells from 4 mice per group, * p<0.05 *vs.* control group (0 concentration of LeTx).

**Figure 2:**
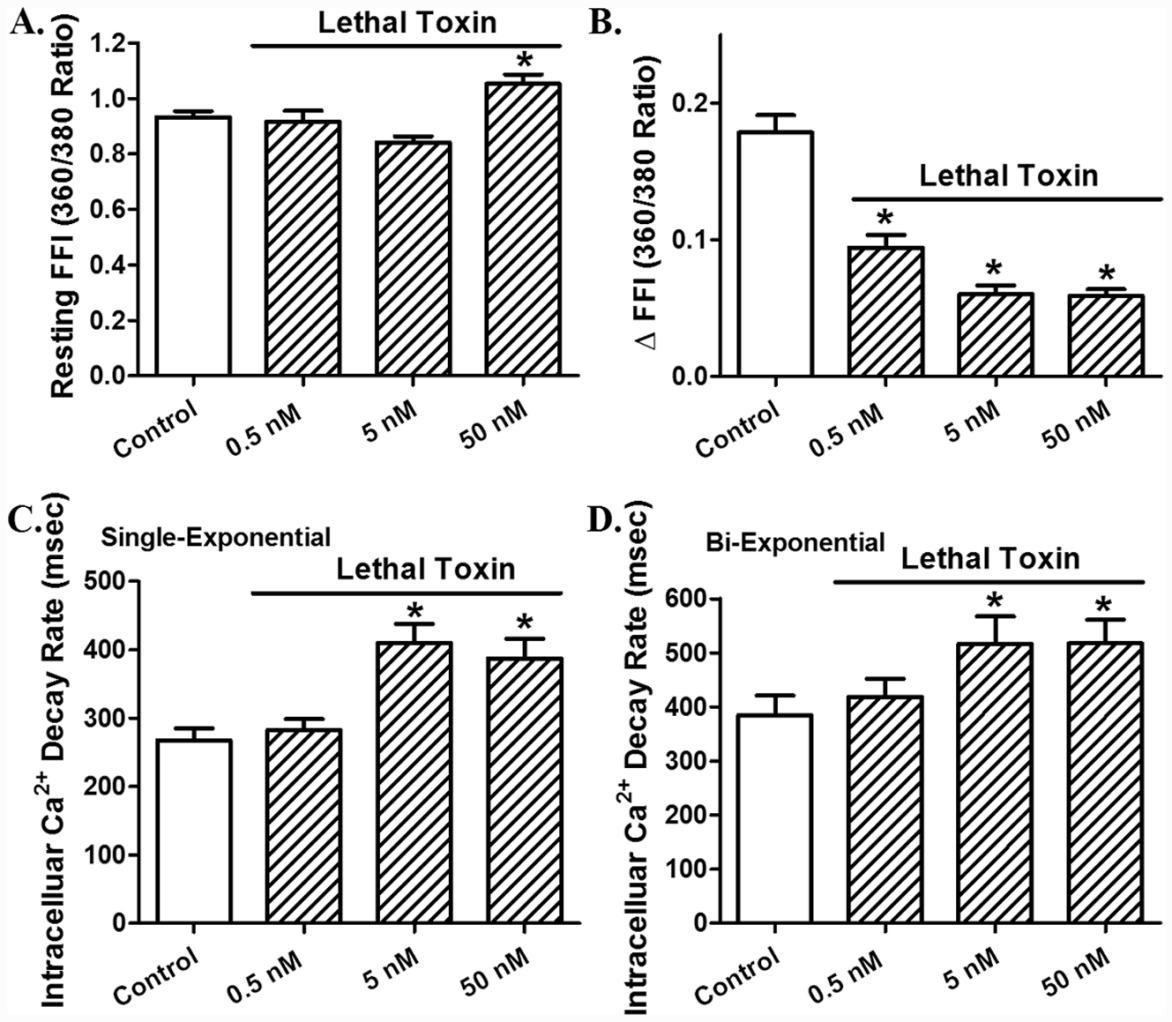
Effect of *in vitro* lethal toxin (0.05-50 nM, 2 hrs) exposure on cardiomyocyte intracellular Ca^2+^ transient property. A: Resting fura-2 fluorescent intensity (FFI); B: Electrically- stimulated rise in FFI (ΔFFI); C: Intracellular Ca^2+^ transient decay rate (single exponential); and D: Intracellular Ca^2+^ transient decay rate (bi-exponential). Mean ± SEM, n = 59 – 60 cells from 4 mice per group, * p<0.05 *vs.* control group (0 concentration of LeTx).

Acute *in vitro* exposure (2 hrs) of lethal toxin displayed a concentration-dependent (0.05 – 50 nM) prolongation in resting cell length and decrease in peak shortening [14] as well as maximal velocity of shortening/relengthening (± dL/dt) in freshly isolated cardiomyocytes. The threshold of response was between 0.05 and 0.5 nM for all these indices. The cell mechanical response of lethal toxin was associated with prolongation in both shortening (TPS) and relengthening (TR_90_) durations although only at the higher concentrations (Fig. 1). To determine whether lethal toxin-induced mechanical responses in cardiomyocyte shortening (PS, ± dL/dt, TPS and TR_90_) were due to altered intracellular Ca^2+^ handling, the influence of lethal toxin on electrically-stimulated intracellular Ca^2+^ transients was examined. Fig. 2 revealed that lethal toxin increased resting intracellular Ca^2+^ level (FFI) only at the highest concentration (50 nM) while eliciting an overt inhibition in intracellular Ca^2+^ rise (ΔFFI) and prolongation in intracellular Ca^2+^ decay (both single and bi-exponential) with a much lower threshold. These data suggest possible involvement of intracellular Ca^2+^-dependent mechanism(s) in acute lethal toxin exposure-elicited cardiomyocyte contractile inhibitory responses.

### In vivo effect of lethal toxin on cardiomyocyte shortening and intracellular Ca^2+^ transients

**Figure 3:**
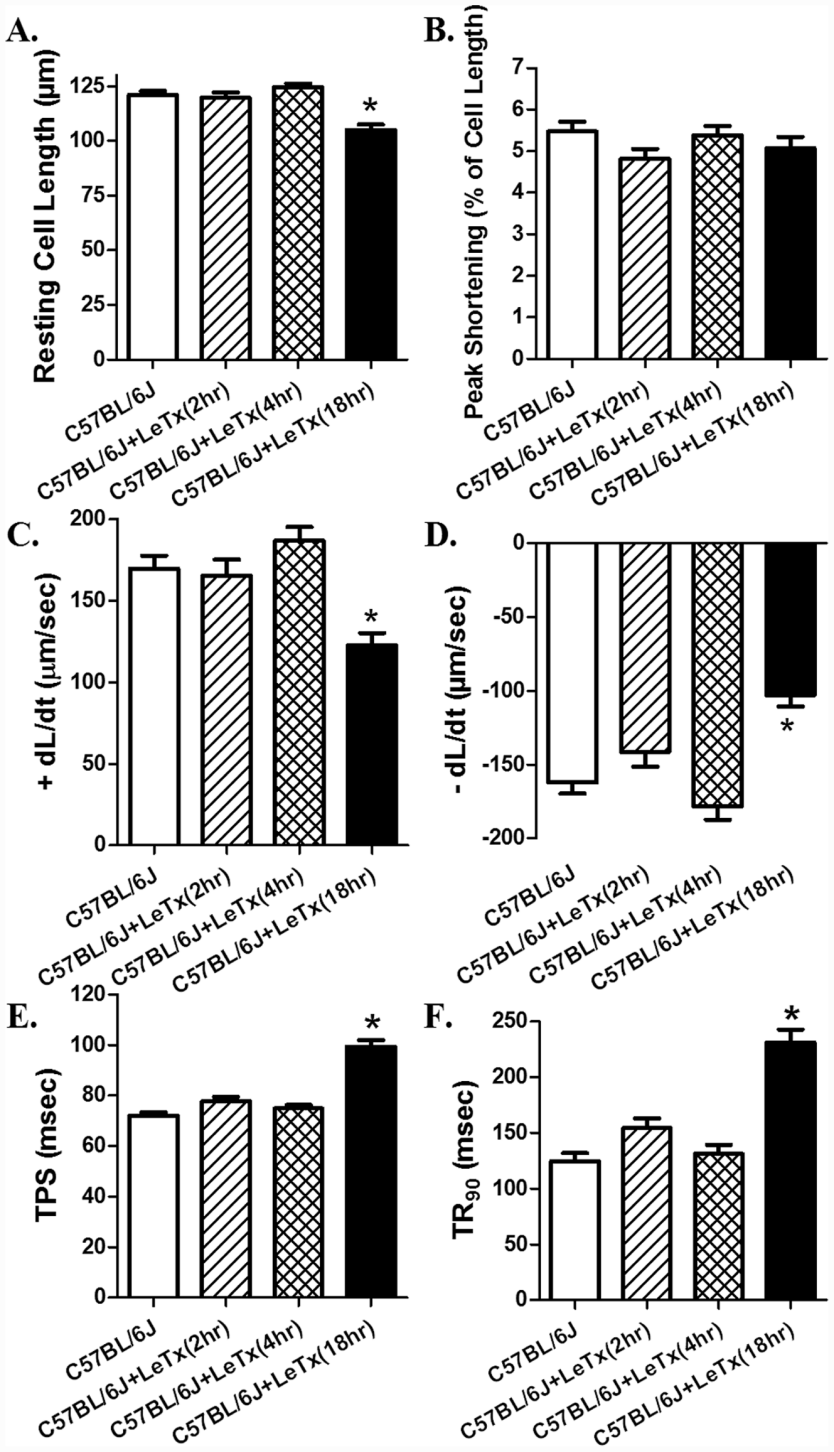
Effect of *in vivo* lethal toxin (LeTx, 2 µg/g b.w., i.p.) exposure for 2, 4 and 18 hrs on cardiomyocyte contractile function. A: resting cell length; B: Peak shortening (normalized to cell length); C: Maximal velocity of shortening (+ dL/dt); D: Maximal velocity of relengthening (- dL/dt); E: Time-to peak shortening (TPS); and F: Time-to-90% relengthening (TR_90_). Mean ± SEM, n = 94–107 cells from 3 mice per time point, * p<0.05 *vs.* C57BL/6J group without LeTx treatment.

**Figure 4:**
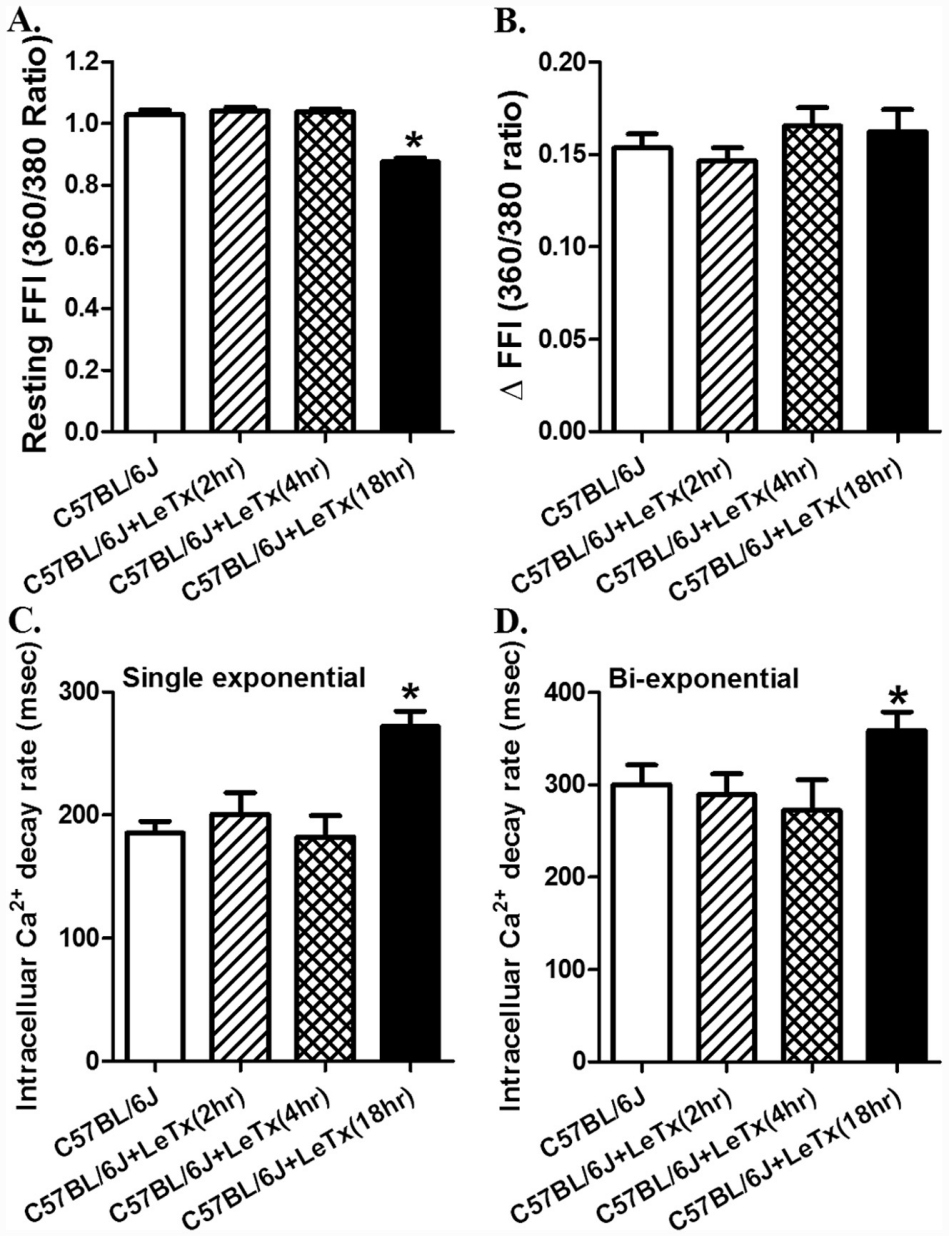
Effect of *in vivo* lethal toxin (LeTx, 2 µg/g b.w., i.p.) exposure for 2, 4 and 18 hrs on murine cardiomyocyte intracellular Ca^2+^ transient property. A: Resting fura-2 fluorescent intensity (FFI); B: Electrically-stimulated rise in FFI (ΔFFI); C: Intracellular Ca^2+^ transient decay rate (single exponential); and D: Intracellular Ca^2+^ transient decay rate (bi-exponential). Mean ± SEM, n = 64–79 cells from 3 mice per time point, * p<0.05 *vs.* C57BL/6J group without LeTx treatment.

Acute *in vivo* exposure of lethal toxin (2 µg/g, b.w., i.p.) triggered a time-dependent mechanical response in cardiomyocyte shortening. Neither 2 nor 4 hrs of lethal toxin exposure was able to elicit any notable effect on resting cell length, PS, ± dL/dt, TPS and TR_90_. Interestingly, longer exposure duration (18 hrs) of lethal toxin significantly decreased resting cell length and ± dL/dt, as well as prolonged TPS and TR_90_ without affecting PS in murine cardiomyocytes (Fig. 3). None of the cell mechanics measured was significantly altered with lethal toxin exposure duration less than 18 hrs. Consistently, *in vivo* lethal toxin exposure failed to overtly affect intracellular Ca^2+^ handling property following 2 and 4 hrs of exposure. Longer duration of lethal toxin exposure (18 hrs) significantly decreased resting intracellular Ca^2+^ level (FFI), and prolonged intracellular Ca^2+^ decay (single and bi-exponential) without affecting rise in intracellular Ca^2+^ (ΔFFI) following electrical stimulus (Fig. 4). Similar to cell shortening, none of the intracellular Ca2+ property was altered with lethal toxin exposure duration less than 18 hrs. These data further consolidated the likely role of intracellular Ca^2+^ handling in lethal toxin exposure (*in vivo*)-elicited cardiomyocyte contractile dysfunctions.

### Role of NADPH oxidase in lethal toxin-induced cardiomyocyte contractile response

**Figure 5:**
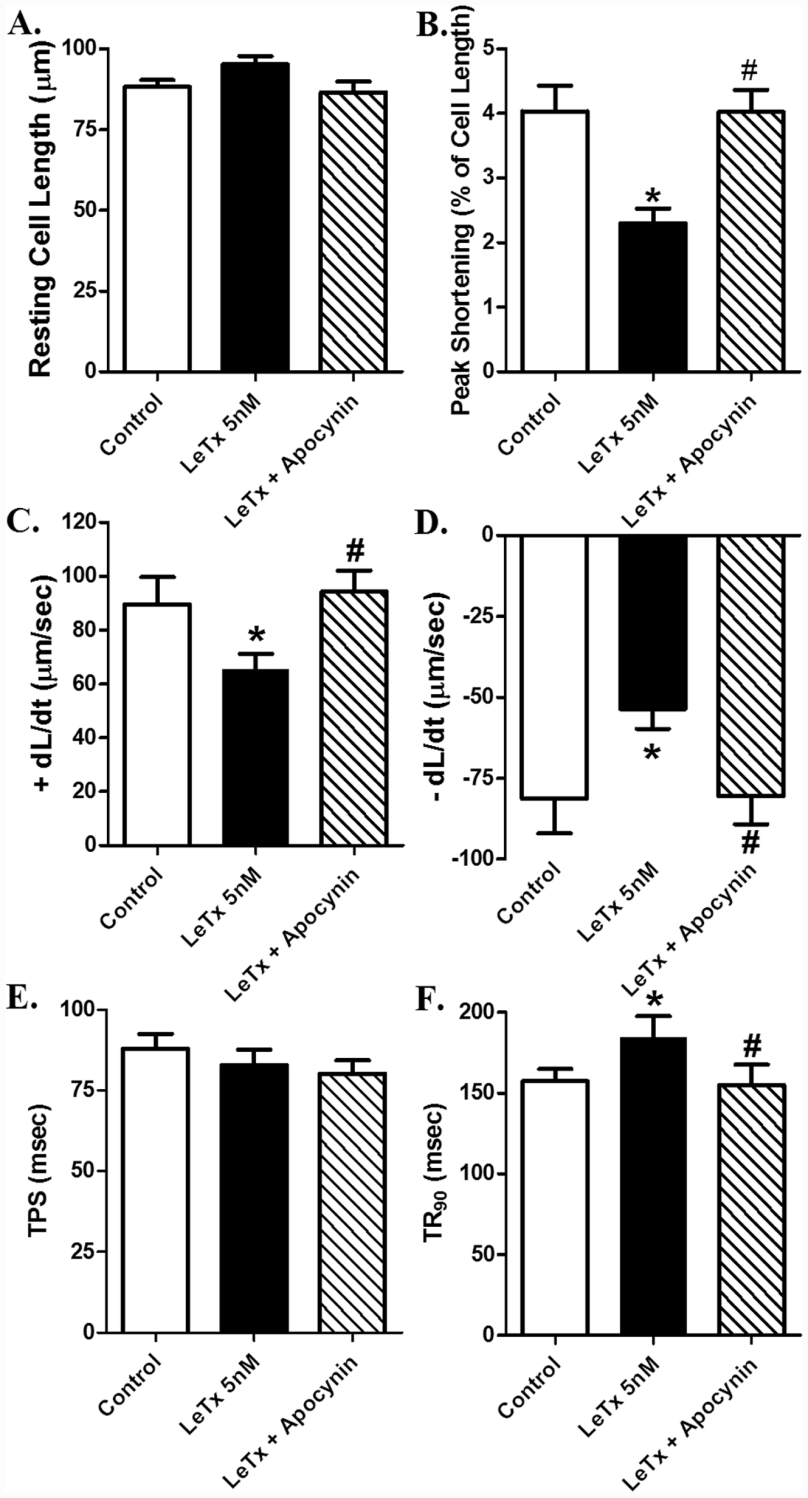
Effect of apocynin (100 nM) pre-incubation on lethal toxin (LeTx, 5 nM, 2 hrs)-induced cardiomyocyte contractile response. A: resting cell length; B: Peak shortening (normalized to cell length); C: Maximal velocity of shortening (+ dL/dt); D: Maximal velocity of relengthening (- dL/dt); E: Time-to peak shortening (TPS); and F: Time-to-90% relengthening (TR_90_). Mean ± SEM, n = 50 cells per group, * p<0.05 *vs.* control group (no drug treatment), #p<0.05 *vs.* LeTx 5 nM group.

To examine the possible mechanism of action behind lethal toxin-induced cardiomyocyte contractile response, the effect of *in vitro* lethal toxin exposure on cardiomyocyte shortening was re-examined in the presence of the NADPH oxidase inhibitor apocynin (100 nM). Apocynin alone had little effect on cell shortening over 2 hrs (data not shown), consistent with our previous report [23]. Interestingly, NADPH oxidase inhibition ablated *in vitro* lethal toxin exposure-induced inhibition in PS and ± dL/dt as well as prolongation of TR_90_ without affecting lethal toxin-elicited action on resting cell length and TPS (Fig. 5). These data favor the notion that NADPH oxidase may play a crucial role in lethal toxin-induced cardiomyocyte contractile dysfunction.

### Role of NADPH oxidase in lethal toxin-induced superoxide generation and cell death

**Figure 6:**
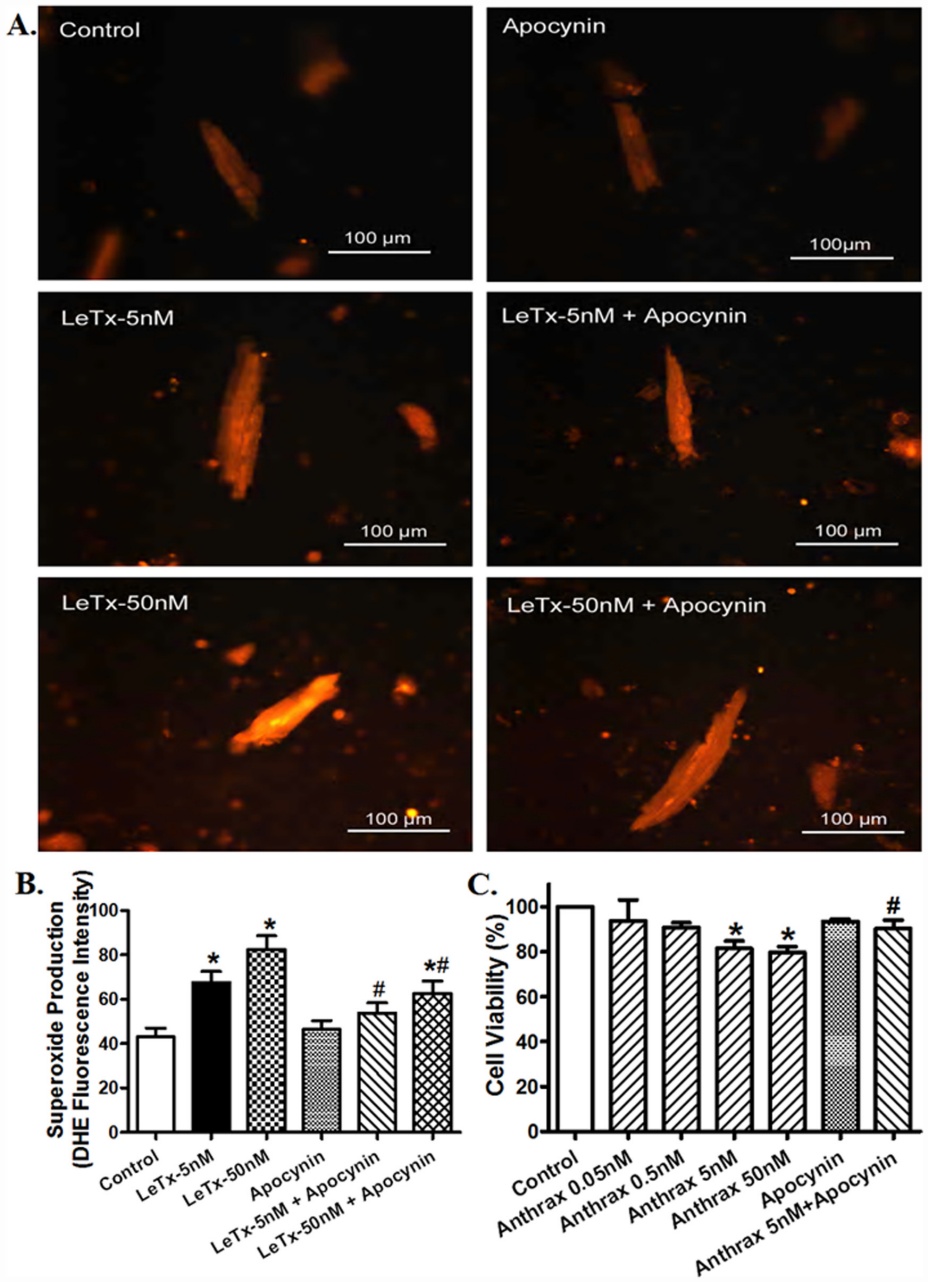
Effect of lethal toxin on O_2_^−^ generation and cell survival using DHE fluorescent probe and MTT assay, respectively, in the absence and presence of the NADPH oxidase inhibitor apocynin (100 nM). A: Representative images depicting DHE fluorescent intensity in murine cardiomyocytes following exposure of various concentrations of LeTx (5 and 50 nM, 2 hrs) in presence and absence of apocynin; B: Pooled data of superoxide production; and C: MTT assay of cell survival following LeTX exposure (0.05 – 50 nM, 2 hrs) in the absence or presence of apocynin. Mean ± SEM, n = 18 and 4 for panels B and C respectively, * p<0.05 *vs.* control group (no drug treatment), #p<0.05 *vs.* corresponding LeTx group in the absence of apocynin.

**Figure 7:**
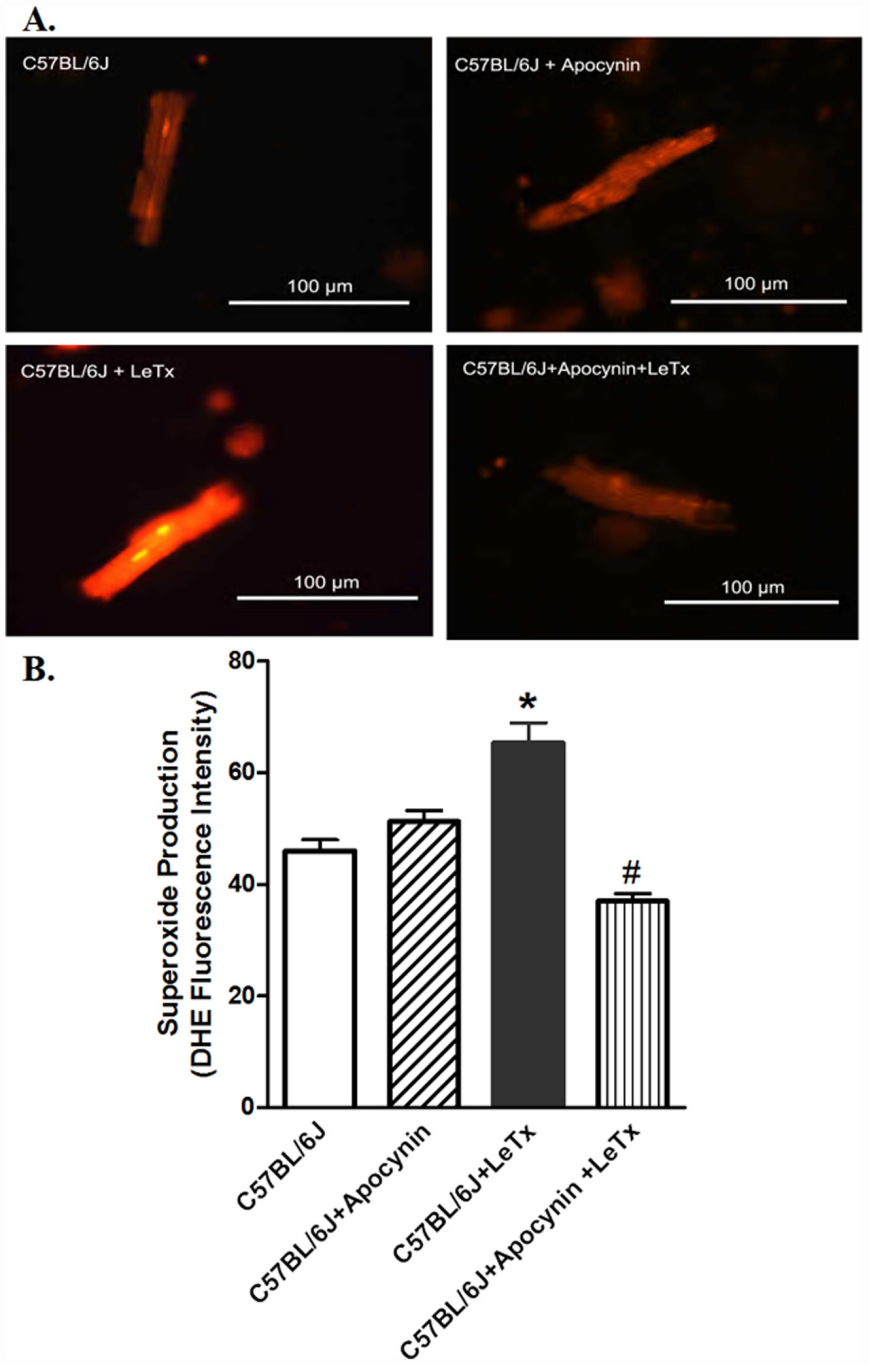
Effect of *in vivo* lethal toxin challenge on O_2_^−^ generation in the absence and presence of the NADPH oxidase inhibitor apocynin pretreatment (80 µg/ml) in drinking water for 6 days. A: Representative images depicting DHE fluorescent intensity in murine cardiomyocytes following exposure of LeTx (2 µg/g b.w., i.p.) for 18 hrs; B: Pooled data of superoxide production. Mean ± SEM, n = 75–100 cells per group. * p<0.05 *vs.* C57BL/6J group, #p<0.05 *vs.* C57BL/6J+LeTx group in the absence of apocynin.

Our result suggested a role of NADPH oxidase in lethal toxin-induced cardiomyocyte contractile dysfunction. To further examine if such NADPH oxidase-mediated mechanism is due to production of superoxide anion and change in cell survival, accumulation of superoxide and cell survival were determined in cardiomyocytes exposed to lethal in the absence or presence of the superoxide generating enzyme NADPH inhibitor apocynin. Fig. 6 exhibits that lethal toxin directly promoted superoxide production and cell death *in vitro*, the effects of which were nullified by apocynin. Our further observation revealed that lethal toxin stimulated ROS generation which was obliterated by apocynin (data not shown). Similarly, *in vivo* examination depicted that pretreatment of mice with apocynin mitigated *in vivo* lethal toxin exposure-induced superoxide production (Fig. 7) and loss of anti-apoptotic proteins (Figure S1) in the heart. These findings supported the notion of a NADPH oxidase-dependent superoxide production and cell death in lethal toxin-elicited cardiomyocyte contractile dysfunction.

### Lethal toxin-induced stress signaling and Ca^2+^ regulatory protein response in vitro

**Figure 8:**
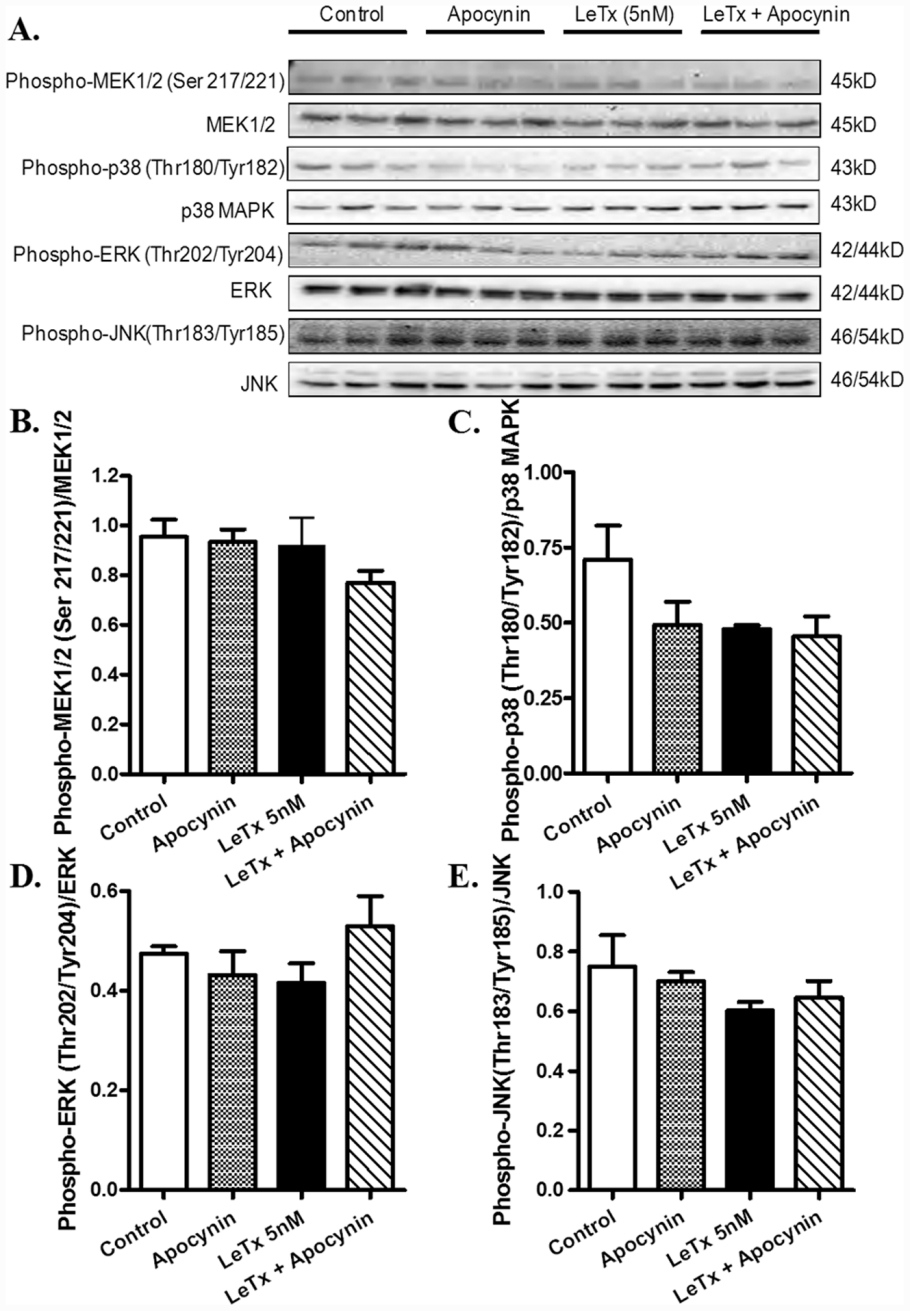
Effect of apocynin on lethal toxin (LeTx)-induced phosphorylation of MEK1/2, p38 MAP kinase, ERK1/2 and JNK. Isolated mouse cardiomyocytes were pre-treated with apocynin (100 nM) for 1 hr followed by incubation of LeTx (5 nM) for 2 hrs. A: Representative Western blot depicting expression of pan and phosphorylated forms of MEK1/2, p38 MAP kinase, ERK1/2, JNK and α-tubulin (loading control) using specific antibodies; B: Phospho-MEK1/2-to-MEK1/2 ratio; C: Phospho-p38 MAP kinase-to-p38 MAP kinase ratio; D: Phospho-ERK1/2-to- ERK1/2 ratio; and E: Phospho-JNK-to-JNK ratio, Mean ± SEM, n = 3 – 4 isolations.

**Figure 9:**
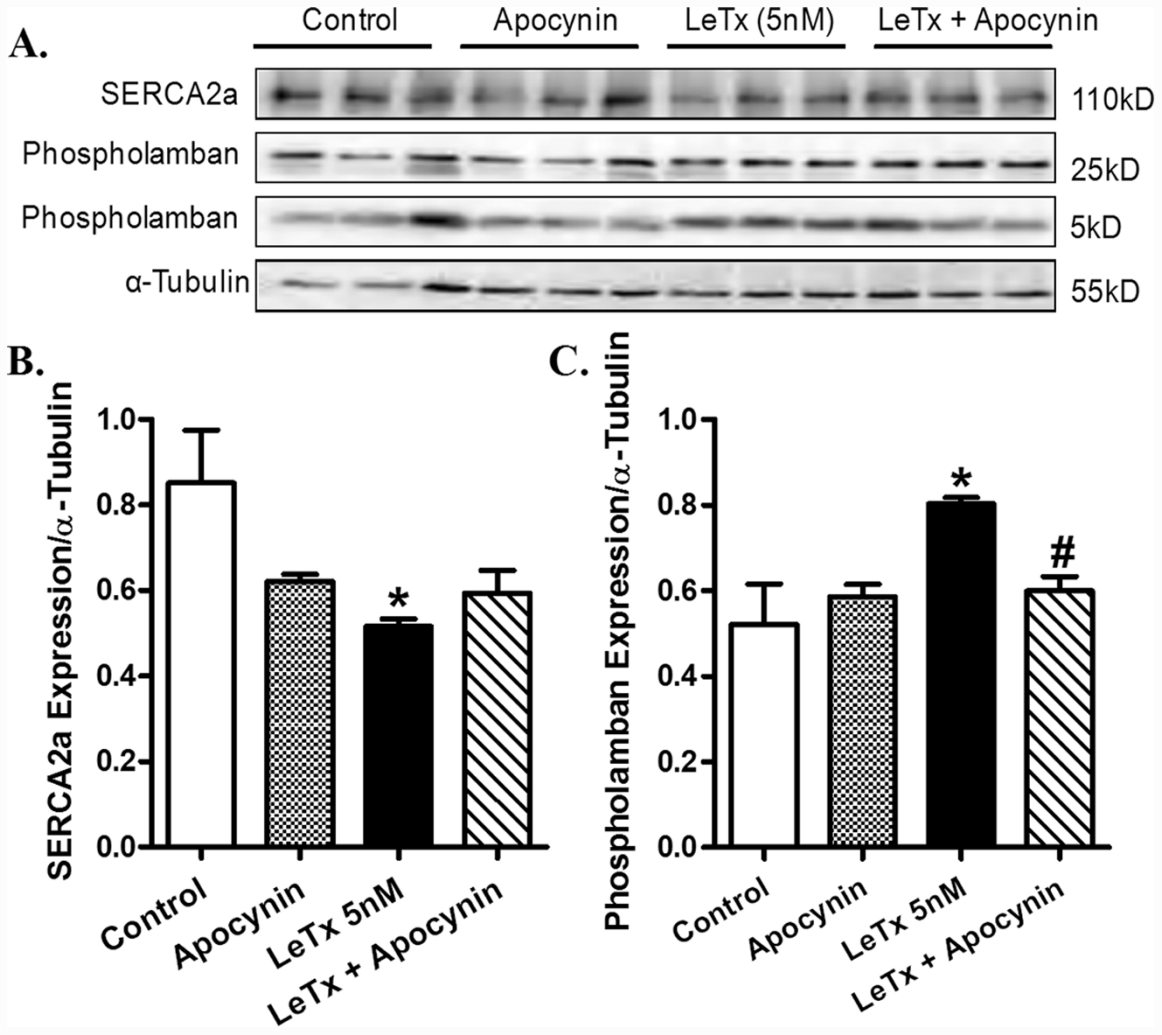
Effect of apocynin on lethal toxin (LeTx)-induced changes in Ca^2+^ regulatory proteins in isolated mouse cardiomyocytes. Isolated mouse cardiomyocytes were pre-treated with apocynin (100 nM) for 1 hr prior to LeTx (5 nM) exposure for 2 hrs. A: Representative Western blot depicting SERCA2a, phospholamban (5 kD or 25 kD subunits) and α-tubulin (loading control) using specific antibodies; B: SERCA2a expression; and C: Phospholamban (combined subunits) expression. Mean ± SEM, n = 3 – 4 isolations, * p<0.05 *vs.* control group (no drug treatment), #p<0.05 *vs.* LeTx 5 nM group.

We further examined the effect of lethal toxin on stress signaling and intracellular Ca^2+^ regulatory proteins as well as the role of NADPH oxidase in lethal toxin-induced responses. Murine cardiomyocytes were exposed to lethal toxin for 2 hrs in the absence and presence of apocynin. Fig. 8 indicated that neither lethal toxin (5 nM, 2 hrs) nor apocynin (100 nM) or both significantly altered the phosphorylation of MEK1/2, p38 MAP kinase, ERK and JNK. *In vitro* exposure of lethal toxin (5 nM. 2 hrs) significantly down-regulated and up-regulated the expression of SERCA2a and phospholamban, respectively, the effects of which were partially restored or ablated by apocynin pretreatment (Fig. 9).

### Lethal toxin-induced stress signaling, Ca^2+^ regulatory protein, ER stress and autophagy response

**Figure 10:**
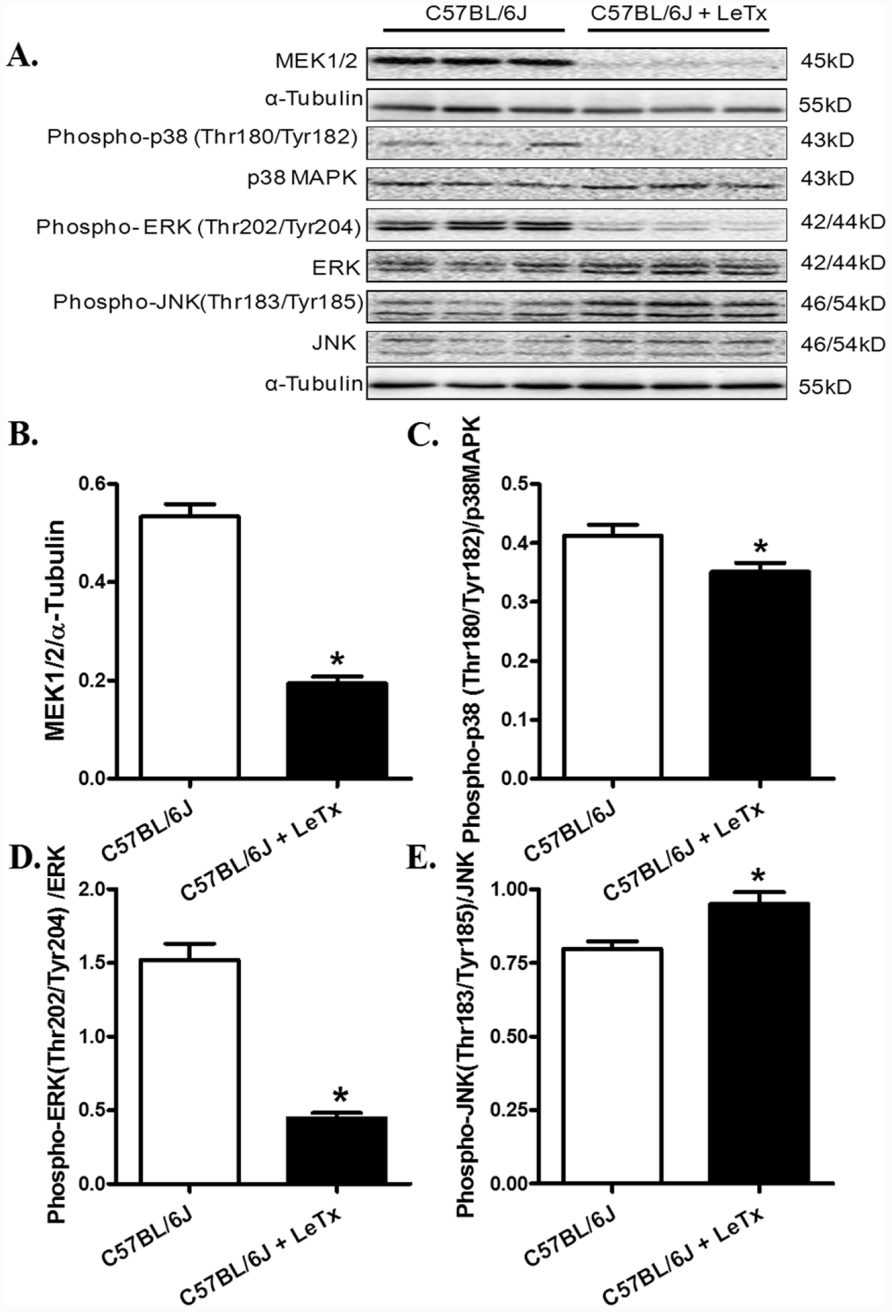
Effect of *in vivo* lethal toxin (LeTX) exposure on MEK1/2, p38 MAP kinase, ERK1/2 and JNK phosphorylation in murine hearts. Adult male C57BL/6J mice were treated with LeTx (2 µg/g b.w., i.p.) for 18 hrs. A: Representative gel blots of MEK1/2, pan and phospho-p38 MAP kinase, pan and phospho-ERK1/2, pan and phospho-JNK and α-tubulin (loading control) using specific antibodies; B: MEK1/2 expression; C: Phospho-p38 MAP kinase-to-p38 MAP kinase ratio; D: Phospho-ERK1/2-to-ERK1/2 ratio; and E: Phospho-JNK-to-JNK ratio. Mean ± SEM, n = 5 – 6 mice, * p<0.05 *vs.* C57BL/6J group without LeTx treatment.

**Figure 11:**
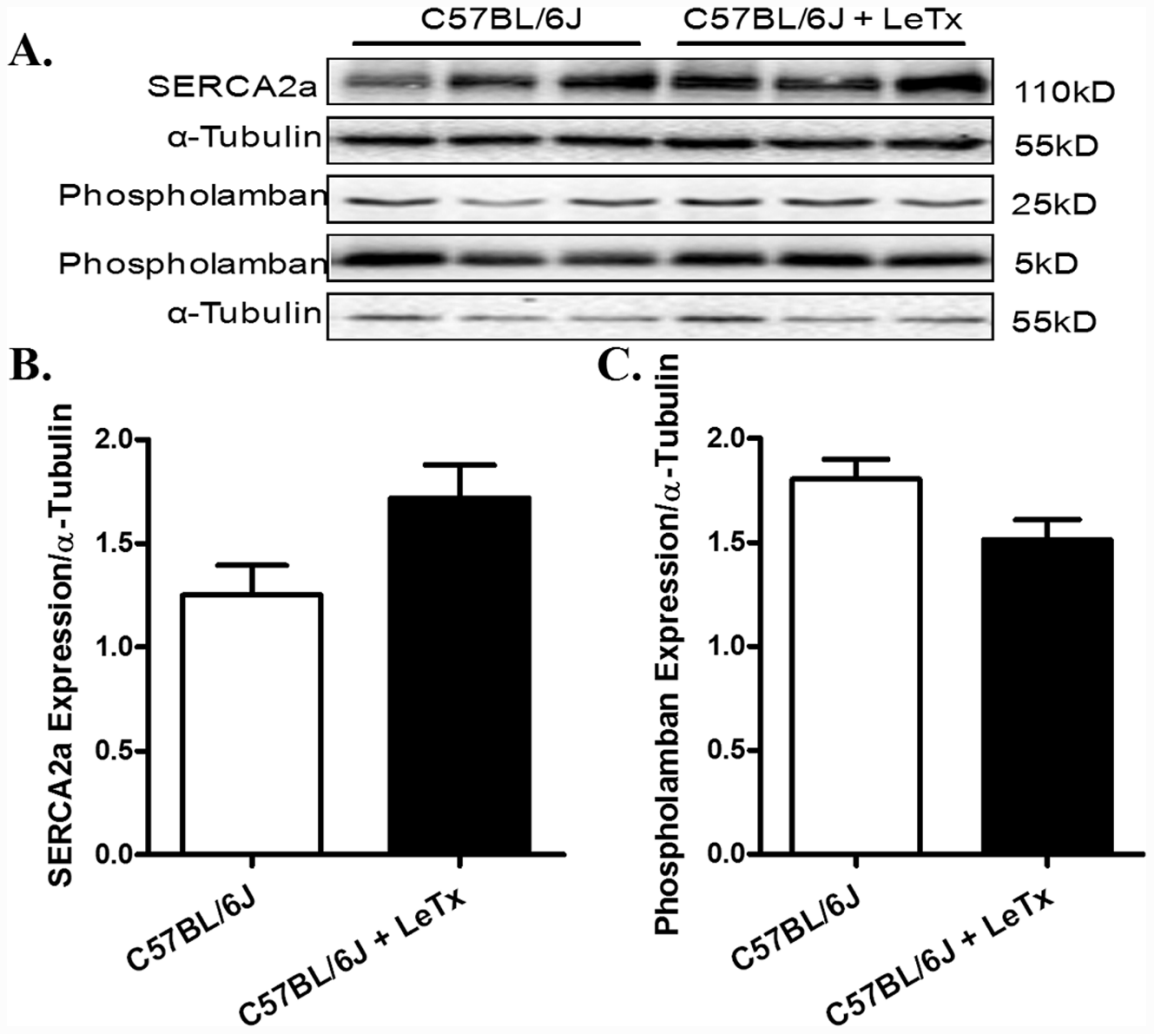
Effect of *in vivo* lethal toxin (LeTx) exposure on intracellular Ca^2+^ regulatory protein expression in murine hearts. Adult male C57BL/6J mice were treated with LeTx (2 µg/g b.w.,i.p.) for 18 hrs. A: Representative gel blots depicting expression of SERCA2a, phospholamban (5 kD and 25 kD subunits) and α-tubulin (loading control) using specific antibodies. B: SERCA2a expression; and C: Phospholamban (both subunits). Mean ± SEM, n = 5 – 6 mice, * p<0.05 *vs.* C57BL/6J group without LeTx treatment.

**Figure 12:**
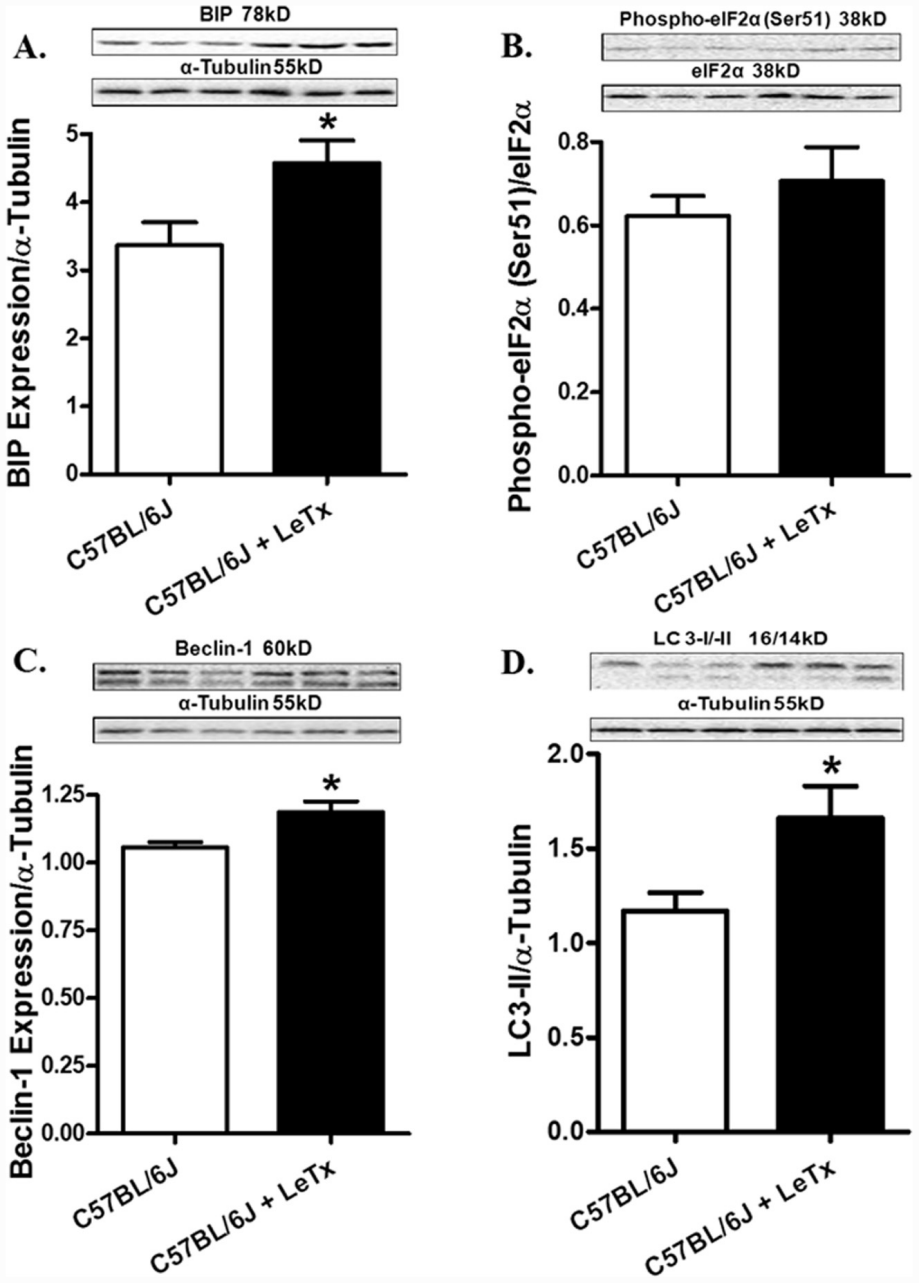
Effect of *in vivo* lethal toxin (LeTx) exposure on ER stress and autophagy markers in murine hearts. Adult male C57BL/6J mice were treated with LeTx (2 µg/g b.w., i.p.) for 18 hrs. A: BIP expression; B: Phospho-eIF2α-to- eIF2α ratio; C: Beclin-1 expression; and D: LC3-II-to-tubulin ratio; Insets: Representative gel blots depicting expression of BIP, pan and phospho-eIF2α, Beclin-1, LC3 and α-tubulin (loading control) using specific antibodies. Mean ± SEM, n = 5 – 6 mice. * p<0.05 *vs.* C57BL/6J group without LeTx treatment.

**Figure 13:**
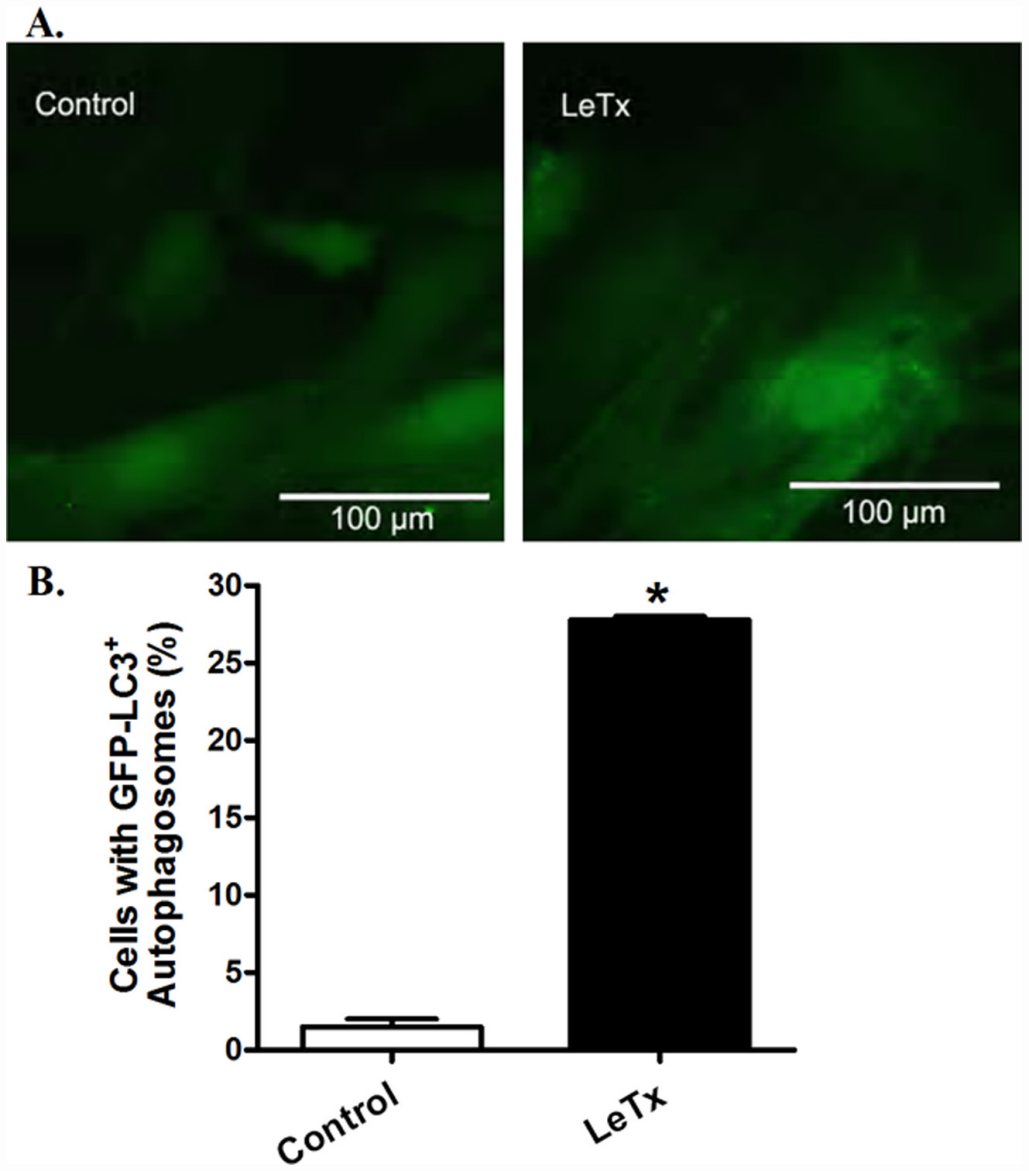
Lethal toxin (LeTx)-induced autophagy in cultured H9c2 cells. H9c2 cells were infected with an adenovirus for 24 hrs to express the LC3-GFP fusion protein. Cells were then exposed to LeTx (100 ng/ml) for 3 hrs. (A) Representative images showing GFP-LC3 puncta in H9c2 cells after LeTx exposure. (B) Percentage of cells with autophagosomes. The cells with 10 or more punctate spots were scored as positive for autophagosomes. Mean ± SEM, n = 100 cells per group from 3 independent experiments, *p<0.05 *vs.* control group in the absence of LeTx.

Evaluation of stress signaling pathways following *in vivo* challenge of lethal toxin (2 µg/g, b.w., i.p.) for 18 hrs revealed overtly downregulated MEK1/2, phospho-MAP kinase and phospho-ERK associated with hyperactivation of JNK in murine hearts (Fig. 10). However, prolonged exposure (18 hrs) of lethal toxin *in vivo* failed to affect the expression of intracellular Ca^2+^ regulatory proteins SERCA2a and phospholamban (Fig. 11). To examine the role of other possible stress signaling mechanism in lethal toxin-induced cardiomyocyte mechanical response, protein markers for ER stress and autophagy were monitored in murine hearts following 18 hrs of *in vivo* exposure of lethal toxin (2 µg/g, b.w., i.p.). Our results revealed that lethal toxin produced subtle although significant upregulation in the expression of the ER stress maker BIP and the autophagy markers Beclin-1 and LC3-II without affecting the phospho-eIF2α-to- eIF2α ratio in murine hears (Fig. 12), suggesting a possible role of autophagy (and a minor one of ER stress) in lethal toxin-induced cardiomyocyte mechanical dysfunction. To further consolidate the lethal toxin-induced autophagy, H9c2 cells were transfected with an adenovirus expressing the LC3-GFP fusion protein prior to the exposure of lethal toxin. Representative images shown in Fig. 13 displayed GFP-LC3 puncta in H9c2 cells following lethal challenge exposure. A greater percentage of cells were found with autophagosomes following lethal toxin treatment.

## Discussion

Data from our current study provided evidence for the first time that *in vitro* and *in vivo* anthrax lethal toxin exposure directly depressed cardiomyocyte contractile function and intracellular Ca^2+^ handling. The lethal toxin-associated cardiomyocyte mechanical depression (reduced peak shortening, maximal velocity of shortening/relengthening, prolonged duration of shortening and relengthening) may be underscored by superoxide production and cell death via a NADPH oxidase-dependent mechanism. These results may suggest a likely role of cardiomyocyte dysfunction in anthrax infection-induced pathophysiological change in the heart.

Although cardiac defects triggered by anthrax toxin seem to depend upon the presence of two toxin components, i.e., lethal toxin (PA+LF) and edema toxin (PA+EF) [1], the two toxins apparently affect cardiac function using distinct mechanisms. Lethal toxin is considered to suppress left ventricular systolic function whereas edema toxin seems to affect heart function via preload regulation [16], [17]. Combinations of lethal toxin and edema toxin in systemic anthrax infection is expected to generate more severe hypotension due to an inability of either the heart or blood vessels to respond to lesions in the other organ. Data from our study support the notion of a direct cardiac depressant effect of lethal toxin at the level of isolated cardiomyocytes. Lethal toxin has been shown previously to suppress myocardial function in a manner reminiscent of fulminant myocarditis [16], [17]. Mice intravenously infused with anthrax lethal toxin were shown to develop malaise and mortality by 60 to 100 hrs [11], [24]. Histopathological findings revealed hypoxic tissue necrosis in liver, marrow, spleen and heart, associated with pleural and peritoneal fluid and edema [16], [24]. Similarly, rodents exposed to lethal toxin died after 2 to 21 hrs due to refractory hypotension, bradycardia, lactic acidosis and pleural effusions [6], [13], [17], [25]. It should be mentioned that either component of lethal toxin (LF and PA) cannot initiate any overt cardiac contractile response, as shown previously [10], [16]. Similarly, neither component of the edema toxin alone is capable of eliciting any effect on ventricular function. Rats receiving intravenous infusion of LF alone survived and showed no symptom of hypotension. In the absence of PA, injection of EF does not cause any symptom in animals [10], [26]. Only combination of PA with LF or EF, but not LF or EF alone, displays cellular toxicity in various cell types such as macrophage cells and T lymphocytes [8], [24].

Several mechanisms may be postulated for lethal toxin exposure-induced cardiomyocyte contractile dysfunction. Our results showed that lethal toxin significantly inhibits cardiomyocyte contractile function as well as intracellular Ca^2+^ transient, suggesting a likely role of intracellular Ca^2+^ homeostasis in lethal toxin-induced inhibition of cardiomyocyte contraction. Our findings of depressed PS and prolonged TR_90_ following lethal toxin exposure are in line with the reduced intracellular Ca^2+^ rise (ΔFFI) and slowed intracellular Ca^2+^ decay or clearing. In general, there is a general agreement in the mechanical and intracellular Ca^2+^ parameters measured between the *in vitro* and *in vivo* lethal toxin studies. The subtle disparity in peak shortening amplitude and intracellular Ca^2+^ rise (ΔFFI) between *in vitro* and *in vivo* lethal toxin exposure may be due to the seemingly discrepant effect of lethal toxin on resting cell length (elongation *in vitro* and shortening *in vivo*) as peak shortening amplitude is normalized to resting cell length. The precise mechanism behind lethal toxin-associated disparity in resting cell length is unknown at this point although difference between *in vitro* and *in vivo* experimental settings may play a role. In our hand, *in vivo* lethal toxin exposure failed to elicit any obvious cardiomyocyte mechanical and intracellular Ca^2+^ handling changes until 18 hrs following treatment. The time-dependent response of *in vivo* lethal toxin exposure is somewhat supported by our earlier report [15]. Our *in vitro* data revealed that cellular machineries responsible for cardiac relaxation such as SERCA and phospholamban were downregulated and upregulated, respectively, in response to lethal toxin exposure. These changes are in line with prolonged cardiac relaxation and delayed intracellular Ca^2+^ decay in response to lethal toxin exposure. SERCA is considered as the single most important machinery to remove Ca^2+^ from cytosolic space (responsible for 92% Ca^2+^ removal) for cardiac relaxation to occur [27]. Our results revealed downregulated SERCA in conjunction with upregulated phospholamban, an endogenous inhibitor of SERCA, following *in vitro* lethal toxin exposure, favoring a likely role of SERCA/phospholamban in lethal toxin-induced intracellular Ca^2+^ dysregulation. Somewhat surprisingly, prolonged *in vivo* exposure (18 hrs) failed to significantly affect expression of SERCA and phospholamban, suggesting presence of compensatory mechanism or time-dependence in lethal toxin-induced response on Ca^2+^ regulatory proteins. Furthermore, although our *in vitro* data do not favor a major role of stress signaling MEK1/2, p38 MAP kinase, JNK and ERK in lethal toxin-elicited cardiomyocyte response *in vitro*, the fact that these stress signaling molecules were significantly down-regulated (MEK1/2, p38 MAP kinase and ERK) or upregulated (JNK) in the *in vivo* setting of lethal toxin exposure suggest a time-dependent response for stress signaling activation. Additional studies using myocardial tissues from anthrax lethal toxin-treated animals at various time points are in need to better elucidate these kinase activity as well as the link between various stress signaling activation and cardiomyocyte mechanical alterations under anthrax lethal toxin infection.

Perhaps the most novel finding from our study was that NADPH oxidase inhibition with apocynin mitigated lethal toxin-induced cardiomyocyte contractile dysfunction, intracellular Ca^2+^ mishandling, intracellular superoxide generation, cell death as well as change in Ca^2+^ regulatory proteins SERCA2a and phospholamban. These data indicate a key role of NADPH oxidase in lethal toxin-induced cardiomyocyte dysfunction. NADPH oxidase has emerged as a key source of reactive oxygen species with diverse cellular functions including anti-microbial defense, inflammation and redox signaling. NADPH oxidase is expressed in the cardiovascular system and is involved in physiological and pathological processes such as the regulation of cardiac contractility, vascular tone, cell growth, migration, proliferation, hypertrophy, apoptosis and matrix deposition [20], [28]. NADPH oxidase is composed of mainly four subunits, two of them are localized in the membrane (gp91^phox^ and p22^phox^) and the others are in the cytosol (p47^phox^, p67^phox^) [29]. Apocynin is known to inhibit p47^phox^ and p67^phox^ translocation and superoxide formation through blockade of sulfhydryl groups thus interrupting the NADPH oxidase enzyme assembly [30]. Several studies have indicated that induction or up-regulation of p47^phox^ and p67^phox^ protein may occur within a short time frame as short as 15 min [20], [31], [32]. Activation of NADPH oxidase, superoxide and reactive oxygen species are known to cause cardiomyocyte contractile dysfunction [20], [28], [33]. Assessment of ER stress and autophagy revealed a significant increase in Bip, Beclin-1 and LC3-II expression following lethal toxin exposure, suggesting possible involvements of ER stress and autophagy. The elevated expression of Beclin-1 and LC3-II following lethal toxin exposure was further substantiated by the overtly increased GFP-LC3 puncta staining in H9C2 cells following lethal toxin challenge. Future study is warranted to elucidate the interplay among NADPH oxidase-induced oxidative stress, ER stress and autophagy behind lethal toxin-induced cardiomyocyte dysfunction.

In conclusion, our study demonstrates that lethal toxin triggers cardiomyocyte contractile dysfunction and intracellular Ca^2+^ mishandling, possibly through NADPH oxidase-mediated superoxide production and cell death. These findings not only reveal a novel role of cardiomyocyte function in anthrax lethal toxin-induced cardiovascular anomalies but also provide new signaling insights for lethal toxin-induced cell injury. The precise mechanism behind anthrax lethal toxin-induced cardiac pathophysiological changes is still unclear. Future studies should focus on its action on cardiac excitation-contraction coupling and membrane ion channels. These approaches will be essential to further our understanding of the pathophysiology and therapeutic strategy of anthrax infection-induced collapse in cardiovascular function.

## Supporting Information

Figure S1 Effect of in vivo lethal toxin (LeTx) exposure on the expression of Bcl-xL, Bcl-2, Bax, Bad, cytochrome C and caspase-12 in murine hearts. Adult male C57BL/6J mice were treated with LeTx (2 µg/g b.w., i.p.) for 18 hrs. A: Representative gel blots of Bcl-xL, Bcl-2, Bax, Bad, cytochrome C, caspase-12 and α-tubulin (loading control) using specific antibodies; B: Bcl-xL; C: Bcl-2; D: Bax; E: Bad; F: Cytochrome C; and G: Caspase-12. Mean ± SEM, n = 4 –5 mice, * p<0.05 vs. C57BL/6J group without LeTX treatment.
(0.20 MB DOC)
