# Supplementary material for: Anthrax Lethal Toxin Suppresses Murine Cardiomyocyte Contractile Function and Intracellular Ca2+ Handling via a NADPH Oxidase-Dependent Mechanism
Source: PLoS One. 2010 Oct 13;5(10):e13335. doi: 10.1371/journal.pone.0013335 (PMC2954163; doi:10.1371/journal.pone.0013335)
Supplement: Figure S1 — Effect of in vivo lethal toxin (LeTx) exposure on the expression of Bcl-xL, Bcl-2, Bax, Bad, cytochrome C and caspase-12 in murine hearts. Adult male C57BL/6J mice were treated with LeTx (2 µg/g b.w., i.p.) for 18 hrs. A: Representative gel blots of Bcl-xL, Bcl-2, Bax, Bad, cytochrome C, caspase-12 and α-tubulin (loading control) using specific antibodies; B: Bcl-xL; C: Bcl-2; D: Bax; E: Bad; F: Cytochrome C; and G: Caspase-12. Mean ± SEM, n = 4 –5 mice, * p<0.05 vs. C57BL/6J group without LeTX treatment. (0.20 MB DOC) [file pone.0013335.s001.doc]

# Anthrax Lethal Toxin Suppresses Murine Cardiomyocyte Contractile Function and Intracellular Ca^2+^ Handling via a NADPH Oxidase-Dependent Mechanism

**Machender R. Kandadi^1^, Yinan Hua^1^, Heng Ma^1^, Qun Li^1^, Shu-ru Kuo^2^, Arthur E. Frankel^2^ and Jun Ren^1^**

**^1^Center for Cardiovascular Research and Alternative Medicine, University of Wyoming College of Health Sciences, Laramie, WY 82071, USA;**

**^2^Cancer Research Institute of Scott & White Memorial Hospital, Temple, TX 76502, USA**

**Supplemental Fig. S1**

**Supplemental Fig. S1:** Effect of *in vivo* lethal toxin (LeTx) exposure on the expression of Bcl-xL, Bcl-2, Bax, Bad, cytochrome C and caspase-12 in murine hearts. Adult male C57BL/6J mice were treated with LeTx (2 µg/g b.w., i.p.) for 18 hrs. A: Representative gel blots of Bcl-xL, Bcl-2, Bax, Bad, cytochrome C, caspase-12 and α-tubulin (loading control) using specific antibodies; B: Bcl-xL; C: Bcl-2; D: Bax; E: Bad; F: Cytochrome C; and G: Caspase-12. Mean ± SEM, n = 4 – 5 mice, * p < 0.05 *vs.* C57BL/6J group without LeTX treatment.

**A.**

**B.**

**C.**

**D.**

**E.**

**F.**

**G.**
